# Supplementary material for: Natural hybridization in heliconiine butterflies: the species boundary as a continuum
Source: BMC Evol Biol. 2007 Feb 23;7:28. doi: 10.1186/1471-2148-7-28 (PMC1821009; doi:10.1186/1471-2148-7-28)
Supplement: Additional File 1 — Hybrids between species of Heliconius and Eueides butterflies: a database. HTML file linking to database of all known wild-caught interspecific hybrid specimens in the Heliconiina, consisting of introductory text, a list of specimens, together with collection data and photographs of the specimens, and links to information about some artificial hybrids and mutants in the group. This is an edited copy of our online database of Heliconius hybrids [102]. To view database, download zip file and extract to a separate folder, then open index.html within that folder. [file 1471-2148-7-28-S1.zip › ethnum01.html]

hybrid ethnum01


---


  
Hybrid between *Heliconius ethilla* *narcaea* and *H. numata
ethra*
  
Brazil
  
© J. Mallet

Return to table of hybrids

To next hybrid
  
To previous hybrid

```
NOTES  
  
No:                      22  
Genus of species 1:      Heliconius  
Species 1:               ethilla  
Subspecies of species 1: narcaea  
Genus of species 2:      Heliconius  
Species 2:               numata  
Subspecies of species 2: ethra  
Sex:                     f  
Country:                 Brazil  
Locality:                Leopoldina  
Year:                    <1908  
Photo no.:               ethnum01  
Named hybrid:            pseudonarcaeus Riffarth ined.  
Collection:              BM  
Collector:               Riffarth  
Author/publication:      Brown 1976: 15c  
Notes:                   F1?
```

**Last updated:** 18 October 2003

---
